# Comparison of uterine fibroids’ growth pattern during pregnancy according to fetal sex: an observational study

**DOI:** 10.1186/s13293-019-0266-2

**Published:** 2019-11-27

**Authors:** Giovanni Delli Carpini, Valeria Verdecchia, Maria Papiccio, Camilla Grelloni, Andrea Ciavattini

**Affiliations:** Obstetrics and Gynecologic Section, Department of Odontostomatologic and Specialized Clinical Sciences, Università Politecnica delle Marche, Via F. Corridoni, 11–60123 Ancona, Italy

**Keywords:** Fibroid, Myomas, Pregnancy, Growth, Obstetric complications, Fetal sex, Male, Female

## Abstract

**Background:**

To investigate the effect of fetal sex on fibroids’ growth during pregnancy according to the hCG serum levels

**Methods:**

Observational study conducted from January 2007 to December 2016 on women with ultrasound identification of uterine fibroids who had a pregnancy within 1 year from diagnosis. The fibroids diameter was determined during the pre-pregnancy ultrasound, early first trimester (5–7 weeks), late first trimester (11–13 weeks), second trimester (19–21 weeks), and third trimester (31–33 weeks). The diameter growth was calculated in each interval between two ultrasounds. The hCG serum levels were determined both in early and late first trimester. The correlation between hCG levels and fibroid diameter was evaluated. Obstetric outcomes collected were gestational weeks at birth and the rate of cesarean section. Neonatal outcomes were birthweight and Apgar score at 1 min.

**Results:**

Eighty-seven of the included women had a male fetus, and 70 had a female fetus. A progressive increase of fibroid diameter was observed from pre-pregnancy to second trimester for both fetal sexes. In third trimester, the mean ± SD fibroid diameter of female fetuses showed a slowdown, while the mean ± SD fibroid diameter of male fetuses continued to grow. Women carrying a female fetus presented a higher fibroid diameter in early first trimester (33.5 ± 13.3 mm vs 27.4 ± 11.0 mm, *p* < 0.01), late first trimester (40.2 ± 13.9 mm vs 34.6 ± 11.7 mm, *p* < 0.01), and second trimester (40.5 ± 14.9 mm vs 34.7 ± 10.3 mm, *p* < 0.01). The hCG serum levels resulted higher in women with a female fetus: 61406 (50554-71760) mU/ml vs 46016 (37160-56744) mU/ml (*p* < 0.01). A positive correlation between hCG levels and fibroid diameter was found both for male and female fetuses (male *r* = 0.77, 95% CI 0.71–0.82, *p* < 0.01 and female *r* = 0.82, 95% CI 0.76–0.86, *p* < 0.01).

**Conclusion:**

Women with female fetus seem to have a higher growth of fibroids up to second trimester of pregnancy. This process may be mediated by the higher serum hCG levels found in women expecting a female fetus.

## Background

Uterine fibroids are the most common benign gynecologic tumors in the reproductive years, with an incidence directly related to age [1–3]. The prevalence of uterine fibroids in pregnancy varies between 3% and 12%, depending upon the trimester of assessment and the size threshold [1–6]. The diagnosis of fibromatosis during pregnancy will probably become more and more frequent due to the progressive elevation of maternal age at the time of first pregnancy and the increasing number of ultrasound scans that are performed during pregnancy Lee HJ, Norwitz ER, Shaw J. Contemporary management of fibroids in pregnancy. Rev Obstet Gynecol. 2010 Winter;3(1):20-7.Zaima A, Ash A. Fibroid in pregnancy: characteristics, complications, and management. Postgrad Med J. 2011 Dec;87(1034):819-28..

Recent evidence shows that fibroids can increase the risk of adverse obstetrical outcomes (such as preterm birth, preterm premature rupture of membranes (pPROM), or breech presentation) with a direct correlation with their number and size [7]. The dimensional increase of uterine fibroids during pregnancy could also influence the incidence of these complications [8].

The growth of uterine fibroids during pregnancy appears to occur more rapidly up to mid-pregnancy, with a peak in the first trimester and stabilization or initial regression in the second half of pregnancy [8]. Numerous mediators that undergo significant variations in the first weeks of pregnancy seem to influence this trend, with a crucial role of hCG. Indeed, a direct correlation between hCG levels and the diameter of the fibroids up to the 12th week has been reported [8] and in vitro studies have confirmed the presence of specific receptors on leiomyomatous cells, which respond to exponential increases in the concentration of hCG [9].

One of the factors that most influences the bio-humoral condition of the first weeks of pregnancy seem to be the fetal sex and, specifically, women with female fetus may present higher values of hCG compared to male fetuses [10, 11].

Thus, the objective of this study was to evaluate the effect of fetal sex on the growth trend of uterine fibroids and to verify the possible correlation with hCG levels.

## Methods

This was an observational study conducted on a cohort of child-bearing age women followed from pre-pregnancy to post-partum. The population presented in this study represents an extension of a cohort analyzed in a previously published paper by our research group [8].

All patients presented an ultrasound diagnosis of uterine fibroids performed at the Obstetrics and Gynecologic Section, Department of Odontostomatologic and Specialized Clinical Sciences, of Università Politecnica delle Marche (Ancona, Italy), from January 2007 to December 2016, with no immediate indication for medical therapy or conservative surgical treatment, and which became pregnant within 1 year from diagnosis.

The first eligibility criterion was the presence of at least one fibroid with a mean diameter higher or equal to 10 mm identified before pregnancy during a transvaginal ultrasound performed for mild gynecological symptoms (abnormal uterine bleeding, pelvic pain, and urinary tract or bowel compression). All women with a spontaneous singleton pregnancy that occurred within 1 year from the initial diagnosis of uterine fibroids constituted the study population.

As exclusion criteria, we considered recourse to in vitro fertilization techniques, diagnosis of multiple pregnancy, miscarriage, or ectopic pregnancy.

All enrolled patients performed routine pregnancy assessments and delivered at our center. Each included patient signed an informed consent to ultrasound execution and data collection. The local ethical committee approved the execution of the present study (Comitato Etico Regionale Marche, CERM Prot. N 2015 0486OR).

As background characteristics, we collected maternal age at pregnancy, BMI (considering pre-pregnancy maternal weight), number of previous pregnancies, smoking, previous oral contraceptive use, previous spontaneous miscarriage, previous cesarean delivery, and previous therapy for fibroids.

All included women underwent five ultrasound examinations: the first in the pre-pregnancy period (within 1 year before the last menstrual period), the second between 5 and 10 completed gestational weeks (early first trimester), the third between 11 and 13 completed gestational weeks (late first trimester), the fourth between 19 and 21 completed gestational weeks (second trimester), and the fifth between 31 and 33 completed gestational weeks (third trimester). During the pregnancy ultrasounds, we also collected the obstetric parameters such as the vitality of the embryo or the fetus, the placental location, and the amount of amniotic fluid.

Ultrasounds were performed with a Voluson 730 PRO (GE Healthcare, Milwaukee, WI, USA) and a 3.5–5.5 MHz probe by the same senior sonographer. We defined uterine fibroids as well-defined round uterine lesions, with heterogeneous echogenicity, shadows at the edge of the lesion or internal fan-shaped shadowing, and circumferential flow around the lesion [12]. We determined three perpendiculars diameters (D1, D2, and D3 in mm), the location (anterior, posterior, right lateral, left lateral or fundal), the site (according to the FIGO classification) [13], and the relationship with the placenta (retroplacental or not retroplacental) for each fibroid. The mean of the three determined diameters (D1, D2, and D3) was considered for analysis purposes.

We defined as “change in fibroid diameter” (∆D) the difference between the final mean diameter (FD) of each fibroid and the starting mean fibroid diameter (SD) in two consecutive ultrasounds (FD–SD). We defined the diameter growth rate (GRD%) of fibroids in two successive ultrasounds by the following formula: GRD% = (100*∆D/SD).

Human chorionic gonadotropin serum levels (hCG) were collected for each patient both during early first trimester (5–7 gestational weeks) and during late first trimester (11–13 gestational weeks), with an ELISA test (VIDAS, bioMerieux; variability coefficient interassay, 5.2%; variability coefficient interassay, 5.6%).

We considered as obstetric outcomes the gestational age at delivery, the rate of preterm birth (less than 37 complete gestational weeks), and the rate of cesarean section.

The following neonatal outcome was collected after birth: sex (male/female), weight, and Apgar score at 1 min.

## Statistical analysis

The statistical software used was SPSS 20 (SPSS Inc., Chicago, IL, USA). The normality of each variable was evaluated by the D’Agostino-Pearson test. Normally distributed variables (age, BMI, fibroid diameter, gestational age at delivery, and neonatal weight) were expressed as arithmetic mean ± standard deviation, while not-normally distributed variables (number of previous pregnancies, GRD%, and hCG levels) were reported as median and interquartile range (IQR). Qualitative variables were expressed as numbers and percentages. The chi-square test, the Mann-Whitney test, or the one-way ANOVA test was used for variable comparison, as appropriate. Correlation between linear variables was evaluated with the Pearson’s *r* coefficient. A multiple regression analysis was conducted considering as dependent variable fibroid diameter at early first trimester ultrasound and late first trimester ultrasound and as independent variables the maternal age, maternal BMI, number of fibroids, FIGO classification, hCG levels at 5–7 weeks, and hCG levels at 11–13 weeks. A *p* value < 0.05 was regarded as a statistically significant.

## Results

**Fig. 1:**
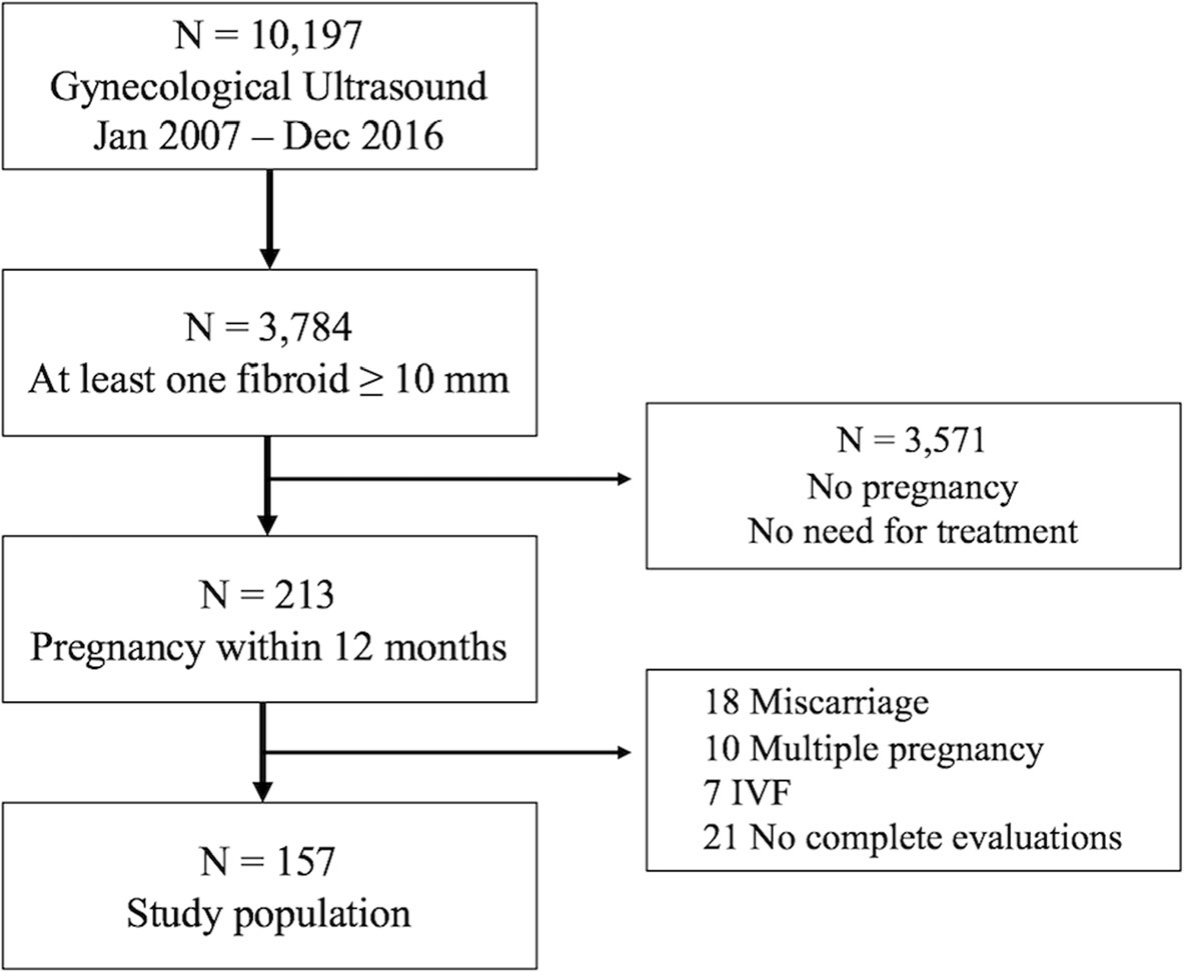
Study flow diagram

During the study period, 10,197 childbearing age women were subjected to a gynecological ultrasound at our institution for mild gynecological symptoms (abnormal uterine bleeding, pelvic pain, and urinary tract or bowel compression). Among these, 3784 (37.1%) were diagnosed with at least one uterine fibroid with a diameter greater than 10 mm. For 2399 (63.4%) women, it was a new diagnosis, while the remaining 1385 were aware that they were affected by uterine fibromatosis. A pregnancy was obtained within 1 year from diagnosis in 213 women, and 157 of them fulfilling the eligibility criteria were considered for the final analysis. More specifically, 18 women were excluded because of spontaneous miscarriage, 10 because of multiple pregnancies, 7 for IVF treatments, and 21 did not complete all the ultrasound evaluations (Fig. 1).

Eighty-seven (55.4%) patients had a male fetus, while 70 (44.6%) had a female fetus. Table 1 compares the socio-demographic features of the group with a male fetus with the group of a female fetus; no difference emerged between the two groups.

**Table 1:** Comparison between the sociodemographic features of patients with male (*n* = 87) and female (*n* = 70) fetuses

Characteristics	Male fetus (*n* = 87)	Female fetus (*n* = 70)	*p*
Age (years)	34.5 ± 5.2	34.3 ± 4.4	0.79
BMI (kg/m^2^)	24.6 ± 3.7	24.5 ± 3.7	0.78
BMI < 18.5	2 (2.3)	2 (2.8)	0.75
BMI 18.5–24.9	47 (54.0)	38 (54.4)	0.91
BMI 25–29.9	30 (34.5)	26 (37.1)	0.87
BMI 30–34.9	8 (9.2)	3 (4.3)	0.38
BMI 35–39.4	0 (–)	1(1.4)	0.93
BMI > 40	0 (–)	0 (–)	(–)
Number of previous pregnancies	1 (0–1)	1 (0–1)	0.83
Nulliparous	32 (36.7)	25 (35.7)	0.97
Smoking	2 (2.3)	6 (8.6)	0.16
Previous oral contraceptive use	23 (26.4)	18 (25.7)	0.93
Previous spontaneous miscarriage	30 (34.5)	25 (35.7)	0.99
Previous cesarean delivery	15 (17.2)	11 (15.7)	0.97
Previous therapy for fibroids	12 (13.8)	10 (14.3)	0.89

Data reported as mean ± SD, median (IQR), or *n* (%) as appropriate *t* test, chi-square test, or Mann-Whitney test as appropriate

At the pre-pregnancy ultrasound, 119 patients (66.1%) presented a single fibroid, and 38 (33.9%) multiple fibroids; the total number of identified lesions was 180. The number, site, and location of fibroids remained the same until the second trimester, allowing the paired comparison between pre-pregnancy and first or second trimester. Table 2 reports the comparison of fibroid characteristics between male and female fetuses: no significant difference was found, except a higher prevalence of anterior fibroids in women with a male fetus (*p* = 0.01).

**Table 2:** Comparison of fibroids characteristics between fetal sexes

Characteristics	Male fetus (*n* = 99 fibroids)	Female fetus (*n* = 81 fibroids)	*p*
Site			
FIGO 7	2 (2.0)	2 (2.5)	0.78
FIGO 6	14 (14.1)	9 (11.1)	0.70
FIGO 5	1 (1.0)	2 (2.5)	0.84
FIGO 4	79 (79.9)	61 (75.3)	0.58
FIGO 3	3 (3.0)	7 (8.6)	0.19
Location			
Anterior	55 (55.6)	29 (35.8)	0.01
Posterior	51 (51.5)	43 (53.1)	0.95
Fundic	4 (4.0)	5 (6.2)	0.74
Right lateral	6 (6.1)	0 (–)	0.07
Left lateral	5 (5.1)	4 (4.9)	0.78

Data are reported as *n* (%). Chi-squared test

There was no difference in fibroid diameter in the pre-pregnancy ultrasound between women with male or female fetuses (*p* = 0.92) (Table 3). The diameter growth analysis showed a significant growth from pre-pregnancy to the second trimester for both fetal sexes (*p* < 0.01), with an increase up to late first trimester (*p* < 0.01), and no difference between late first and second trimester (*p* = 0.95 for male fetuses and *p* = 0.90 for female fetuses) (Table 3). Women with a female fetus presented a significantly higher fibroid diameter both during first and second trimester with respect to women with a male fetus (*p* < 0.01) (Table 3). The mean ± SD fibroid diameter during third trimester was higher than all previous ultrasound for male fetuses (39.1 ± 13.2 mm). In women carrying a female fetus, mean ± SD fibroid diameter during the third trimester (37.0 ± 11.9 mm) was lower than the late first and second trimester (Table 3).

**Table 3:** Diameter growth trend of fibroid dimensions from pre-pregnancy to second trimester according to fetal sex

Ultrasound	Diameter (mm)		
	Male fetus (*n* = 99)	Female fetus (*n* = 81)	*p*
Pre-pregnancy	23.7 ± 11.7	23.5 ± 12.8	0.92*
Early first trimester	27.4 ± 11.0	33.5 ± 13.3	< 0.01*
Late first trimester	34.6 ± 11.7	40.2 ± 13.9	< 0.01*
Second trimester	34.7 ± 10.3	40.5 ± 14.9	< 0.01*
Third trimester	39.1 ± 13.2	37.0 ± 11.9	0.29
*p*	< 0.01^	< 0.01^	–

Data are reported as mean ± SD. *t test. ^One-way ANOVA

No difference in fibroid diameter between women with or without previous therapy for fibroids emerged both during the early first trimester (34.0 ± 12.0 mm vs 29.5 ± 12.4 mm, *p* = 0.08) and the late first trimester (39.5 ± 11.5 mm vs 36.9 ± 14.0 mm, *p* = 0.37).

The diameter growth rate (%) between pre-pregnancy and early first trimester was significantly higher in women with a female fetus, in comparison to a male fetus (33 (8–68)% vs 25 (5–47)%, *p* = 0.04), while no difference emerged between the early first and late first trimester (17 (0–38)% vs 23 (0–64)%, *p* = 0.16) or between first and second trimester (0 (− 12–7)% vs 2 (0–12)%, *p* = 0.07). The diameter growth rate was different between females and males between second and third trimester (6 (4–9)% vs 6 (− 7–8)%, *p* = 0.02).

The median (IQR) level of hCG in male fetus was 30309 (3856–47604) mU/ml, significantly lower than the female fetus hCG level of 34403 (2432–62350) mU/ml (*p* = 0.04). A positive correlation with the gestational weeks was found for both the fetal sexes (male *r* = 0.77, 95% CI 0.71–0.82, *p* < 0.01 and female *r* = 0.82, 95% CI 0.76–0.86, *p* < 0.01).

The hCG levels of women with or without previous therapy for fibroids were not different: 5–7 weeks (6816, IQR 1533-16314 vs 2785, IQR 914-8240, *p* = 0.06) and 11–13 weeks (54420, IQR 45852-63615 vs 49548, IQR 39287-62007, *p* = 0.41).

**Fig. 2:**
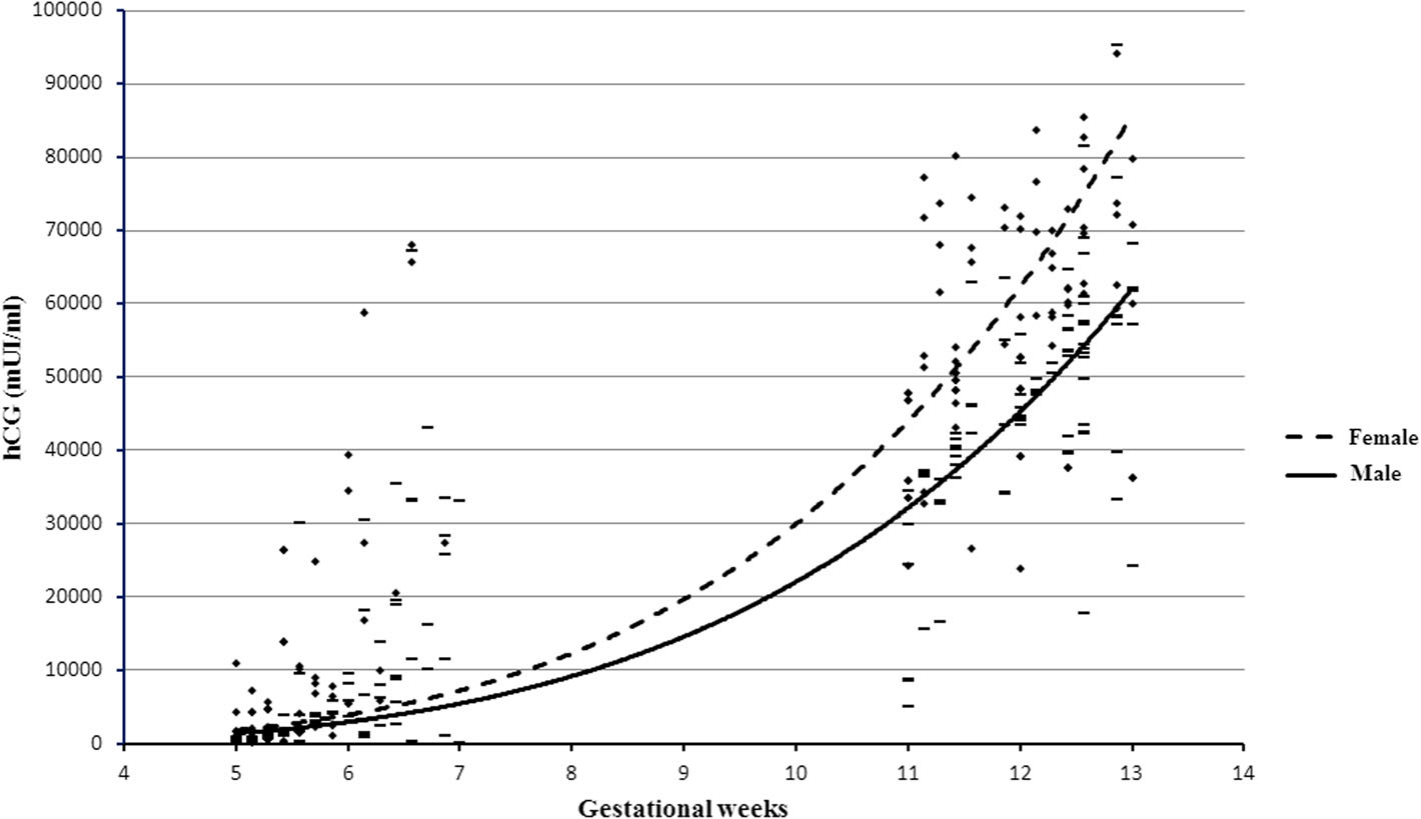
Comparison between hCG levels between male and female fetuses. Black diamond for females and en dash for males

No significant differences were found between the male (3856, IQR 1010-9799 mU/ml) and female (2432, IQR 914-10121 mU/ml) median (IQR) levels of hCG during early first trimester (5–7 weeks) (*p* = 0.74). Conversely, a significant difference was found between during late first trimester (11–13 weeks): 46016 (37160-56744) mU/ml vs 61406 (50554-71760) mU/ml, *p* < 0.01, when the pregnancies with female fetus reached higher levels (Fig. 2).

**Fig. 3:**
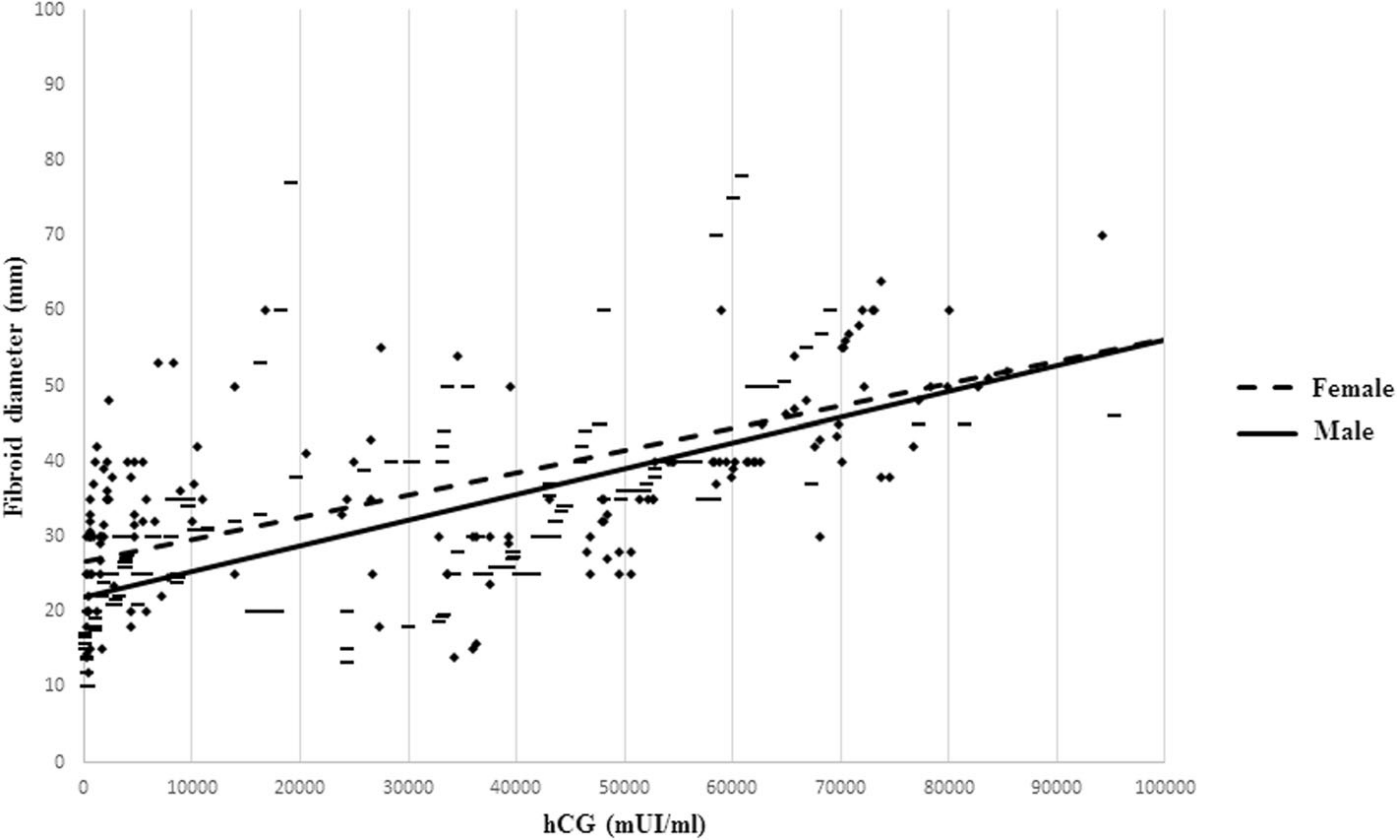
Correlation between hCG levels and fibroid diameter in the two fetal sexes. Black diamond for females and en dash for males

A positive correlation between hCG levels and fibroid diameter during first trimester was found both for male (*r* = 0.68, *p* < 0.01) and female fetuses (*r* = 0.65, *p* < 0.01) (Fig. 3). More specifically, during the early first trimester, the Pearson’s *r* coefficient for female fetuses was 0.69 (95% CI 0.55–0.79, *p* < 0.01) and for male fetuses was 0.68, (95% CI 0.56–0.78, *p* < 0.01). During the late first trimester, the Pearson’s *r* coefficient for females was 0.87 (95% CI 0.81–0.92, *p* < 0.01), and for males was 0.75 (95% CI 0.65–0.83, *p* < 0.01). No difference between correlation coefficients was found during the early first trimester (*p* = 0.94), while in late first trimester the *r* coefficient for females was significantly higher than males (*p* = 0.02).

At the multiple regression analysis, only female sex and hCG values at 5–7 weeks or 11–13 weeks were independently associated with fibroid diameter at early first trimester ultrasound and late first trimester ultrasound (Table 4). Maternal age, BMI, number of fibroids, and FIGO classification were excluded from the model.

**Table 4:** Multiple regressions of factors associated with fibroid diameter at early first trimester and late first trimester

Factor	Early first trimester ultrasound		Late first trimester ultrasound	
	*r*	*p*	*r*	*p*
Fetal sex (female)	0.18	0.02	0.29	0.01
hCG 5–7 weeks	0.53	< 0.01	0.53	< 0.01
hCG 11–13 weeks	0.30	< 0.01	0.68	< 0.01

No difference in gestational age at delivery (38.9 ± 1.6 weeks vs 39.0 ± 1.9 weeks vs *p* = 0.85), rate of preterm birth (6.9% vs 8.6%, *p* = 0.92) or rate of cesarean section (23.0% vs 24.3%, *p* = 0.99) emerged between women with male or female fetuses. The mean ± SD neonatal weight of male fetuses was 3351 ± 504 gr, and of female was 3197 ± 552 gr (*p* = 0.09). The median (IQR) Apgar was 9 (9–9) for males and 9 (9–9) for female (*p* = 0.82).

## Discussion

From the analysis of our data, we noted a growth of uterine fibroids during the first and second trimester of pregnancy for both fetal sexes, even if fibroids in women expecting a female presented larger dimensions both during the first and second trimester. The diameter growth rate was higher in early pregnancy for female fetuses, and both fetal sexes presented a slowdown of fibroid growth up to the second trimester. In male fetuses, the fibroid growth continued until third trimester (even if with a slower rate), while in female fetuses, a reduction in fibroid diameter was noted during third trimester.

In the first trimester, when the difference between males and females was more evident, we noticed a significantly higher hCG serum level in patients with female fetus rather than patients with a male fetus, with a direct correlation between hCG increase and fibroid diameter.

This trend of growth is in line with the literature; indeed, Benaglia et al. observed in 2014 a significant growth of fibroids in the first period of pregnancy in patients who underwent ART procedure [14], and De Vivo et al. reported in 2011 that 71.4% of fibroids increase in size between first and second gestational period [15].

The significant growth rate observed in the first weeks of pregnancy seems to be related to the variation of the hCG serum levels in the same gestational period. This hypothesis is supported by laboratory data that showed the presence of hCG receptors on the fibroid cells [8] and from the clinical observation of a direct correlation between the hCG levels and the growth of fibroids during the first trimester of pregnancy [7].

To date, it is widely documented that the values of hCG are influenced by fetal sex. Yaron et al. reported that in patients who underwent to ART procedures, from the 14th and the 20th day post-fertilization, women attending a female fetus had higher levels of hCG than women attending a male (*p* < 0.002) [10]. Adibi et al. also found a significantly higher hCG level (*p* < 0.0001) in patients with a female fetus rather than patients with a male fetus both during the first and the second trimester on a wide population (1.1 million) of women subjected to screening [11]. The results of our study are mostly in line with those reports, and, more specifically, we did not find any difference in hCG serum levels until the seventh week of pregnancy, but the values of female fetuses were significantly higher between 11 and 13 gestational weeks. Since in our series hCG serum levels did not differ between males and females until the seventh week of pregnancy, it is possible to speculate that different mediators may influence fibroid growth in the first weeks of pregnancy, and the role of hCG may become predominant only in the late first trimester, where hCG reach higher levels, and there is a significant difference between males and females.

The different growth trend observed between males and females during third trimester (progressive increase in dimensions for males and reduction for females) may not be related only to hCG serum levels. Indeed, other hormones (estrogens, progesterone, and testosterone), placental hormones (human relaxin RLNH1), and angiogenic markers (Fms-like kinase 1 and placental growth factor (PlGF) that present differences between fetal sexes may play and additional role on fibroid growth during pregnancy.

Our results are strengthened by a large number of included women and by the early and close monitoring of fibroid characteristics. Moreover, the two subgroups of women with male and female fetus were comparable for all the socio-demographic and sonographic features.

In our series, the discrepancy of fibroid growth between the two fetal sexes was not associated with a different incidence of adverse obstetric outcomes. This lack of association could be explained by the fact that a significant part of the patients included in the study had a single myoma of small size, under the threshold for an increased risk of adverse obstetric outcomes. Furthermore, it is important to underline that the genesis of obstetric diseases is a multifactorial event, not attributable only to the presence of uterine fibroids.

A potential limitation of our study is the sonographic method of evaluation of diameter of fibroids, with the inevitably ultrasound-related degree of imprecision. We tried to overcome this limitation by using a standard technique for fibroid diagnosis and measuring. For these reasons, the determination of fibroid diameter and the evaluation of diameter increase could be affected by an inevitable risk of inaccuracy.

In conclusion, uterine fibroids seem to grow rapidly both during first and second trimester of pregnancy, with faster growth in women expecting a female fetus. This difference could be explained by the direct correlation between hCG serum levels and fibroid diameter found in these patients. After second trimester, the growth continues for male fetuses until the third trimester but it stops for female fetuses.

The early determination of fetal sex (e.g., through noninvasive prenatal tests) could be useful in patients with fibroids and female fetus for a more accurate counseling about the risk of growth of uterine fibroids and the possible onset of adverse obstetric outcomes.

These patients with a higher risk could be initiated to more intensive screening programs, with monitoring of fibroids dimensions, in order to more effectively prevent, diagnose, and handle eventual complications. However, future studies are needed to evaluate the risk of adverse obstetric outcomes related to the growth of fibroids, also in populations with multiple and larger lesions.

## Data Availability

The datasets analyzed are available from the corresponding author on reasonable request.
